# Toward New Therapeutic Mechanisms in Bipolar Disorder: Analog Investigation of Self-Compassion and Nonattachment to Self

**DOI:** 10.3389/fpsyg.2018.01848

**Published:** 2018-09-27

**Authors:** Yan Yang, Kathryn Fletcher, Richard Whitehead, Greg Murray

**Affiliations:** ^1^Centre for Mental Health, Swinburne University of Technology, Hawthorn, VIC, Australia; ^2^Department of Psychological Sciences, Swinburne University of Technology, Hawthorn, VIC, Australia

**Keywords:** bipolar disorder, self-concept, self-compassion, nonattachment to self, therapeutic mechanism

## Abstract

There is growing interest in psychological processes that might be targeted in treatments for bipolar disorder (BD). One such process is a vulnerability at the level of self-concept, characterized by presence of, and fluctuations between positive and negative self-concept. The aim of the present study was to advance this literature by investigating the role of two emerging meta-cognitive processes – self-compassion and nonattachment to self – which have potential to therapeutically modulate this unstable self-concept in BD. Using an analog design, it was hypothesized that both variables would mediate the relationship between bipolar tendencies and psychological distress in a general population sample. Participants (*N* = 372 Australian university students) completed self-report measures of manic and depressive tendencies, self-compassion, nonattachment to self and psychological distress. To investigate the specificity of the two hypothesized mediators, a better-researched psychological variable – rumination – was also included in mediation analyses. Bivariate analyses found tendencies toward mania and depression to be associated with diminished self-compassion and nonattachment to self, while both psychological processes were negatively associated with psychological distress. Mediation analyses showed, as expected, self-compassion and nonattachment to self mediated the relationship between bipolar tendencies and psychological distress after controlling for the effects of rumination. The present findings add incrementally to this literature by demonstrating that two meta-cognitive processes – self-compassion and nonattachment to self – act as mediators, and may be modifiable mechanisms linking bipolar vulnerability to negative mood outcomes. Future research should tackle longstanding conceptual issues in this domain, including the relationship between contents of self-concept (an established focus of BD research) and the person’s meta-cognitive approach to their self-concept (the focus here).

## Introduction

Bipolar disorder (BD) is characterized by extreme mood variability and debilitating psychosocial dysfunction (Goodwin and Jamison, 2007). Adjunctive psychosocial treatments for BD are broadly effective, but modest effect sizes encourage further research into psychological mechanisms that may be open to therapeutic modulation (Frank et al., 2000; Alloy et al., 2005; Lam et al., 2005). Emerging research suggests that one such mechanism is at the level of the self-concept: Coexisting positive and negative self-concept (and its fluctuating prominence) is implicated in the extreme mood states that define the disorder (Power, 2005).

Data from qualitative (Jamison, 1995; Inder et al., 2008) and quantitative studies (Ashworth et al., 1985; Bentall et al., 2005; Knowles et al., 2007; Ak et al., 2012) support the notion that self-concept in BD is characterized by the presence of, and variations between, polarized favorable and unfavorable traits and attributes. For instance, Ak et al. (2012) showed that relative to healthy controls, those with BD reported greater levels of both positive (e.g., grandiosity) and negative (e.g., underdeveloped self) self-related schemas. Literature concerning self-esteem in BD depicts its highly unstable nature fluctuating between inflation of confidence during mania and demeaning self-worth during depression (Knowles et al., 2007; Park et al., 2014). This line of research suggests that a vulnerability at the level of self-concept may contribute to the onset, development and maintenance of BD (Nilsson et al., 2010; Kyrios et al., 2016).

Drawing from third wave psychological therapies, the present project sought to investigate two novel psychological processes – self-compassion and nonattachment to self – that may have potential to ameliorate the self-related vulnerabilities of BD. Both processes can be characterized as meta-cognitive, relating not to the contents of self-concept in BD, but to the person’s subjective approach to their self-concept.

Self-compassion involves approaching oneself with gentle kindness and perceiving difficulties as part of a larger human experience (Neff, 2003; Gilbert, 2009); absence of self-compassion is associated with a self-judgmental attitude, the tendency to over-identify with negative experiences and feeling isolated by suffering (Neff, 2003). Self-compassion therapies have demonstrated efficacy in multiple psychological disorders, including social anxiety disorder (Koszycki et al., 2016), major depressive disorder (Krieger et al., 2016), eating disorders (Braun et al., 2016), and borderline personality disorder (Soler, 2015). Only one study to date has investigated self-compassion in BD, reporting significantly lower levels of self-compassion when compared with healthy controls (Døssing et al., 2015). Drawing from the theoretical framework proposed by Gilbert et al. (2007) which was further elaborated by Lowens (2010), positive and negative evaluations of self-concept in BD hinge critically on the perceived social rank of superiority, and inferiority. As such, through disengaging from social comparison and promoting a mentality of social affiliation, self-compassion may help regulate the over-activated evaluative system directed at self-concept in BD.

Derived from the Buddhist worldview, nonattachment to self refers to releasing rigid attachment to self-concept, through recognizing the fact that ‘self’ is a mental construct, highly contingent on external stimuli (McWilliams, 2014). nonattachment is considered in contemplative tradition as a powerful pathway to increase wellbeing, contentment and connectedness with common humanity (Sahdra et al., 2010). The opposite of nonattachment, that is, attachment (a key source of suffering in Buddhism) engenders fixation with desirable and downplaying undesirable aspects of self-concept, ultimately leading to psychopathology (Sahdra et al., 2010; Vago and Silbersweig, 2012; Shonin et al., 2016). Attachment in this regard critically differs from the well-established attachment concept in western psychology, as the latter is generally understood in the context of secure (or insecure) internal working models (e.g., the person’s perception of how he or she relates to other people and the world) (Sahdra and Shaver, 2013). In Buddhist psychology, attachment inevitably leads to disappointment, distress or even psychopathology, because the attached objects are merely mental constructs that are impermanent and often do not match with the physical reality. Consequently, nonattachment is deemed to be the key remedy to the suffering.

A well-established psychological model of BD depicts a similar process insofar as vulnerability to mood episodes arise from excessive self-directed appraisals of internal and external stimuli (e.g., racing thoughts may be interpreted as high intelligence) and subsequent maladaptive coping behaviors as attempts to regulate these appraisals (Mansell et al., 2007). Drawing links between attachment to self from the Buddhist perspective and the process of regulating self-directed appraisals in BD (Mansell et al., 2007), the potential clinical utility of cultivating nonattachment to self in BD may occur via two pathways: (1) dampening the over-active self-relevant appraisals, and/ or (2) circumventing the tendency for the individual to engage in maladaptive coping behaviors to manage dysregulated perceptions of self.

Instead of addressing the problematic contents of self-concept in BD (viz., the highly polarized and unstable evaluative self-concept), it may be therapeutic to encourage people with BD to adopt a new subjective approach on a meta-cognitive level, to the self-concept. The aim of the present study was to investigate two examples of such an approach: self-compassion, referring to a kind and caring attitude; and nonattachment to self, referring to a style of de-emphasizing the importance of the self as an entity.

The potential therapeutic impact of these variables was investigated in an analog design, with the prediction that both would statistically mediate the relationship between quantified vulnerability to BD and psychological distress in a general population sample. The core mediation hypotheses were tested in analyses that included a third construct – rumination – to investigate the specificity of the proposed mediation pathways, and to control for a better-researched psychological process (e.g., Kim et al., 2012).

## Materials and Methods

### Participants

Participants were first year psychology students participating for course credit at Swinburne University of Technology in Australia. The sample comprised 372 participants (79.8% of female; *M* = 35.54 years, *SD* = 10.71 years). Sixty-six percent were employed and 19% were studying full-time. Approximately 70% obtained an educational degree equivalent or higher than a Diploma. Ethical approval for the study was granted by Swinburne University Human Research Ethics Committee.

### Material and Measures

Measures were administered online via a secure survey platform (Qualtrics).

### Vulnerability to BD

Vulnerability to BD in the general population is most commonly quantified as two correlated but separable dimensions of mania and depression proneness (Bullock and Murray, 2014). Here, the two bipolar traits were measured with the 7 Up 7 Down Inventory (7U7D; Youngstrom et al., 2013), a brief (14-item) version of the General Behavior Inventory (GBI; Depue et al., 1981). Items rated on a four-point scale (0 = never or hardly ever; 3 = very often or almost constantly) include “Have there been times when you have felt that you would be better off dead?” for trait depression (T-Depression), and “Have there been times lasting several days or more when you felt you must have lots of excitement, and you actually did a lot of new or different things?” for trait mania (T-Mania). In the development paper, sound construct validity (e.g., discriminant and convergent validity), and internal consistency (Cronbach’s α between 0.81 and 0.95) were reported, with clear separation of manic and depressive traits (Youngstrom et al., 2013). Internal consistency was high in the present sample (Cronbach’s α = 0.82 and α = 0.92 for T-Mania and T-Depression, respectively).

### Self-Compassion

Self-compassion was measured with the Self-Compassion Scale (SCS; Neff, 2003). The SCS comprises 26 items, designed to measure overall levels of self-compassion. Items rated on a five-point scale (1 = almost never; 5 = almost always) include “When I’m going through a very hard time, I give myself the caring and tenderness I need”. Neff (2003) reported sound psychometric properties of the SCS including high internal consistency (Cronbach’s α = 0.92) and convergent validity. The SCS also showed good cross-cultural validity and reliability (Neff et al., 2008). In a recent paper, Neff et al. (2017) reported good psychometric properties of the SCS as a reliable measure of overall self-compassion. The variable of interest in the present study is the total score of the SCS. Internal consistency was excellent in the present sample (Cronbach’s α = 0.94).

### Nonattachment to Self

nonattachment to self was measured with the nonattachment to Self Scale (NTS; Whitehead et al., unpublished), designed to measure the degree of dispassion/nonattachment to self-concept. Item generation for the NTS involved extensive reviews of Buddhist literature and consultation with experts in the field. A preliminary set of 64 items rated on a 7-point scale (1 = strongly disagree; 7 = strongly agree) was reduced through removal of ambiguous/unclear items and confirmatory factor analysis. The final single-factor 7-item scale demonstrated good internal consistency in the original sample (Cronbach’s α = 0.84). Preliminary evidence of construct validity comes from expected patterns of correlations with other constructs (e.g., mindfulness for convergent validity; depersonalization for discriminant validity). Internal consistency of the NTS was high in the present sample (Cronbach’s α = 0.87).

### Rumination

Rumination was measured with the 10-item Ruminative Response Scale (RRS; Treynor et al., 2003), designed to measure repetitive thoughts about negative feelings, their causes, and associated meanings (Nolen-Hoeksema, 1991). Items rated on a 4-point scale (1 = almost never; 4 = almost always) include “analyse recent events to try to understand why you are depressed”. The variable of interest in the present study is the total RRS score (RRS-T), which has shown good internal consistency (Cronbach’s α = 0.88) (Miranda et al., 2013). Internal consistency was also adequate in the present sample (Cronbach’s α = 0.83).

### Psychological Distress

Psychological distress was measured with the Depression Anxiety Stress Scale-21 (DASS-21; Lovibond and Lovibond, 1995). The DASS-21 is a short form of the original 42-item DASS, designed to measure stress, depression and anxiety. Items rated on a 4-point scale (0 = did not apply to me at all; 3 = applied to me very much, or most of the time) include “I felt I was close to panic”. Whilst DASS is commonly used to measure three separate emotional states, use of the total score to assess psychological distress is appropriate (Lovibond and Lovibond, 1995) and has been adopted in previous BD research (Hubbard et al., 2016). The DASS total score (DASS-T) showed adequate psychometric properties in the development paper (Lovibond and Lovibond, 1995), including excellent internal consistency (Cronbach’s α = 0.95; Hubbard et al., 2016). Internal consistency was good in the present sample (Cronbach’s α = 0.93).

### Statistical Analyses

Mediation hypotheses were tested by analysing whether the effect of the independent variable on the dependent variable were through third variables (mediators). In contrast to investigating mediation hypotheses singly, multiple mediator models (Preacher and Hayes, 2008) were used here as they permit the estimation of unique effects of individual mediators, increase power, and reduce Type I error. Multiple mediator models identify confidence intervals using a bootstrapping technique that involves repeated re-samplings from the obtained dataset to generate an estimated sampling distribution of the mediation effects (Preacher and Hayes, 2008). In the current study, tendencies toward mania (T-Mania) and depression (T-Depression) were the independent variables in two separate mediating analyses. Psychological distress (DASS-T) served as the dependent variable in both analyses. Self-compassion (SCS), nonattachment to self (NTS), and rumination (RRS-T) were included as mediating variables.

## Results

### Descriptive Statistics and Bivariate Correlations

The overall level of BD vulnerability is similar to that observed in other non-clinical samples (Tang-Smith et al., 2015). Bivariate analyses satisfied preliminary criterion for multiple meditational analyses, with significant associations in expected directions (**Table 1**). For example, both mania proneness (T-Mania; *M* = 11.45, *SD* = 3.57) and depression proneness (T-depression; *M* = 11.92, *SD* = 4.27) negatively correlated with self-compassion (SCS; *M* = 83.48, *SD* = 16.43) and nonattachment to self (NTS; *M* = 32.25, *SD* = 7.70), and positively correlated with rumination (RRS-T; *M* = 21.51; *SD* = 5.76) and psychological distress (DASS-T, *M* = 10.43, *SD* = 9.76). The two proposed mediators, self-compassion and nonattachment to self, were strongly positively correlated.

**Table 1:** Summary of bivariate correlations for study variables.

Measures		1	2	3	4	5
T-Mania	1					
T-Depression	2	−0.38^∗∗^				
SCS	3	−0.16^∗∗^	−0.64^∗∗^			
NTS	4	−0.10^∗^	−0.51^∗∗^	0.73^∗∗^		
RRS-T	5	0.36^∗∗^	0.53^∗∗^	−0.36^∗∗^	−0.34^∗∗^	
DASS-T	6	0.29^∗∗^	0.60^∗∗^	−0.57^∗∗^	−0.50^∗∗^	0.24^∗∗^

*N = 372. T-Mania, Trait Mania subscale; T-Depression, Trait Depression subscale; SCS, Self-Compassion Scale; NTS, Nonattachment to Self Scale; RRS-T, Ruminative Response Scale (total score); DASS-T, Depression Anxiety Stress Scale 21-item (total score). ^∗∗^p < 0.01, ^∗^p < 0.05.*

### Hypothesis Testing

#### Trait Mania as a Predictor of Psychological Distress

**FIGURE 1:**
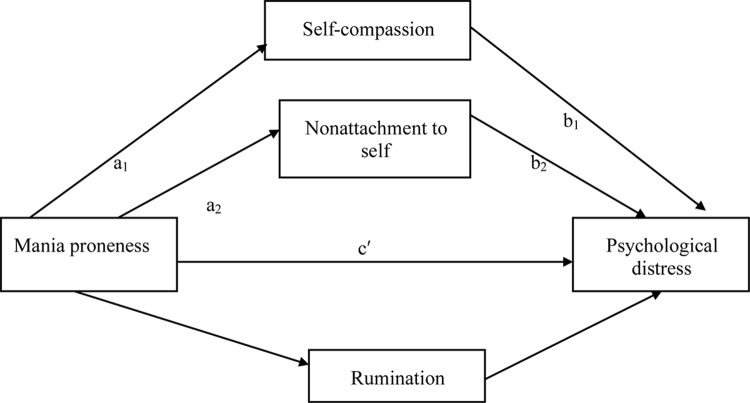
Proposed mediation model examining mania proneness, self-compassion, nonattachment to self, rumination, and psychological distress.

The overall mediation model assessing the indirect effect of mania proneness on psychological distress was significant, explaining approximately 38% of the variance in psychological distress, *F* (4, 367) = 58.65, *p* < 0.001 (**Figure 1**). All three mediators showed significant mediating effects. Specifically, the a paths were significant, showing that greater manic tendency was associated with lowered self-compassion, *SE = 0.24, p < 0.01*, lowered nonattachment to self, *SE = 0.11, p = 0.0486* and higher rumination, *SE = 0.08.11, p < 0.001*. The b paths were significant, showing that lowered self-compassion, *SE = 0.04, p < 0.001*, lowered nonattachment to self, *SE = 0.08, p = 0.02*, and higher rumination, *SE = 0.08, p < 0.01*, were associated with more severe psychological distress. Taken together, the indirect path of manic tendency to psychological distress through self-compassion, nonattachment to self, and rumination was significant *B* = 0.38, *SE* = 0.09, *p* < 0.001, 95% *CI* [0.18, 0.52].

#### Trait Depression as a Predictor of Psychological Distress

**FIGURE 2:**
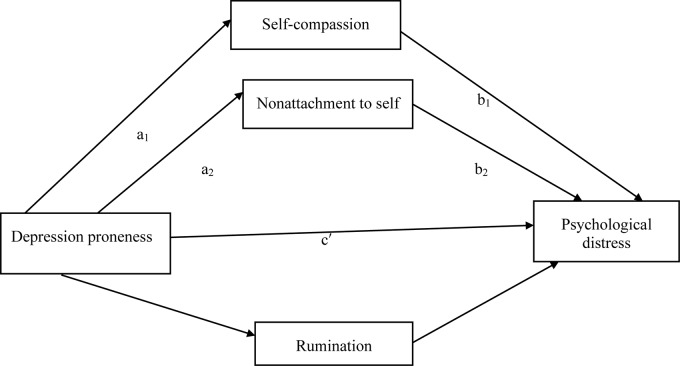
Proposed mediation model examining depression proneness, self-compassion, nonattachment to self, rumination, and psychological distress.

As above, the overall model assessing the indirect effect of depression proneness on psychological distress was significant, explaining approximately 42% of the variance in psychological distress, *F* (4,367) = 68.46, *p* < .001 (**Figure 2**). Self-compassion and nonattachment to self but not rumination showed significant mediating effects. The a paths were significant, showing that greater depressive tendency was associated with lowered self-compassion, *SE = 0.15, p < 0.001*, lowered nonattachment to self, *SE = 0.08, p < 0.001* and higher rumination, *SE = 0.06, p < 0.001*. The b paths were significant for the two proposed mediators, showing that lowered self-compassion, *SE = 0.04, p < 0.001*, lowered nonattachment to self, *SE = 0.07, p = 0*.0467 were associated with more severe psychological distress. However, the b path for rumination was not significant, *SE = 0.08, p = 0.08*. Take together, the indirect path of depressive tendency to psychological distress through self-compassion and nonattachment to self was significant *B* = 0.42, *SE* = 0.11, *p* < 0.001 *CI* [0.36, 0.79].

## Discussion

Motivated by identifying new modifiable psychological targets for the vulnerable self-concept in BD, the present study sought to investigate the role of two emerging constructs, self-compassion and nonattachment to self in BD tendencies. As hypothesized, both constructs significantly mediated the relationship between bipolar tendencies (both mania and depression proneness) and psychological distress. Effects of the hypothesized mediators were net of the variance explained by the better-researched construct of rumination. Bivariate associations will be briefly reviewed, before considering the implications of novel mediation effects identified in the findings, and future research steps.

Consistent with prior reports of diminished self-compassion in BD (Døssing et al., 2015), the current study found that self-compassion strongly associated with depression tendency, and moderately with mania tendency. Low self-compassion has been demonstrated as a robust vulnerability factor in unipolar depression (Krieger et al., 2016). Our results extend this evidence, to suggest that a lack of self-compassion is a vulnerability factor in bipolar depression and this vulnerability may also extend to mania proneness although to a lesser degree.

This is the first study to examine self-compassion as a mediating factor between the two self-reported traits of BD vulnerability (mania- and depression-proneness) and psychological distress, highlighting the importance of this construct as a potential therapeutic mechanism in BD. The present findings are consistent with the growing impact of self-compassion on other psychological disorders including anxiety disorder (Koszycki et al., 2016) and borderline personality disorder (Soler, 2015). Gilbert et al. (2007) suggest that self-evaluation for individuals with BD is linked with a highly sensitive social rank mentality, contributing to extreme mood states. As such, therapeutically self-compassion in BD may assist with down-regulating evaluations directed at self-concept through dampening social competitiveness, and instead fostering a more productive mentality of social affiliation.

The present study is also the first to investigate the role of nonattachment to self in self-reported BD proneness The negative associations between bipolar tendencies and nonattachment to self suggest that at the level of self-concept, vulnerability to BD (particularly vulnerability to bipolar depression) may be characterized by an over-attachment to self-concept. An over-attachment to self-concept in BD that is fragmented and contradictory as shown in existing literature (Power, 2005; Inder et al., 2008; Potter, 2013), may activate a process of excessive self-directed appraisals and subsequent behaviors to regulate the appraisals as suggested by Mansell et al. (2007), ultimately escalating extreme mood states.

The mediating effect of nonattachment to self in the relationship between bipolar tendencies and psychological distress may provide a new therapeutic target in BD. In Buddhist tradition, nonattachment to self, a quality of releasing attachment to self-concept, is a key remedy to psychopathology and a fruitful pathway to wellbeing (Sahdra et al., 2010). Taken together, it is plausible to speculate if individuals with BD cultivate nonattachment to self, ongoing vulnerability to future mood episodes may be reduced.

Psychopathology research has seen a resurgence of interest in targeting psychological vulnerabilities at the level of the self-concept. Much remains unknown, however, about how to best conceptualize, measure and ameliorate such vulnerabilities (Leitan, 2016). In contrast to previous research emphasizing the contents of self-concept (Leitan, 2016), the present findings suggest that self-compassion and nonattachment to self may be promising new therapeutic targets in BD. Whilst the two constructs share conceptual overlap, they may potentially improve psychological functioning via fostering more adaptive relationships (yet with important nuanced differences) with self-concept: self-compassion is through cultivating a compassionate relationship with the self; nonattachment to self is through dampening the over-attachment to self-concept. This focus is consistent with third wave therapeutic approaches (e.g., mindfulness-based interventions) that seek to enhance meta-awareness and acceptance of internal stimuli as they are (Hunot et al., 2013).

## Limitations

The study had several limitations. First and foremost, statistical mediation in a cross-sectional convenience sample quantified by their degree of self-reported vulnerability to BD is simply a first step in investigating putative therapeutic mechanisms at the level of self-concept in BD. As part of a large clinical trial, our group is currently testing whether therapy-related improvements in self-compassion and nonattachment to self correlate with improvements in quality of life and symptoms in late stage BD (Fletcher et al., 2018). Second, self-compassion and nonattachment to self were assessed by self-reported measures, susceptible to response biases. For instance, self-reported assessments of Buddhist constructs may reflect participant aspirations rather than acquired qualities (Grossman, 2011). Finally, while the self-compassion variable was measured via a well-validated and commonly used scale, the nonattachment to self variable as the other proposed mediator was assessed by a novel scale. The psychometric properties of this scale are currently being established (Whitehead et al., unpublished), and this requires further investigation in a clinical sample of individuals with BD.

## Conclusion

The current study suggests that self-compassion and nonattachment to self mediate the relationship between bipolar tendencies and psychological distress. Study findings contribute to the growing research into self-concept in BD as well as psychological mechanisms underlying BD broadly. The study represents the first investigation of two novel constructs to potentially target vulnerability at the level of the self-concept in BD. Future research should utilize clinical samples to investigate the potential role of self-compassion and nonattachment to self in BD.

## Ethics Statement

This study was carried out in accordance with the recommendations of The National Statement on Ethical Conduct in Human Research (2007), Swinburne University Human Research Ethics Committee with written informed consent from all subjects. All subjects gave written informed consent in accordance with the Declaration of Helsinki. The protocol was approved by the Swinburne University Human Research Ethics Committee.

## Author Contributions

YY managed the literature searches, designed the study, collected and analyzed the data, and wrote the first draft of the manuscript. KF and GM as supervisors of YY contributed to all stages of the study including literature searches, study design and analyses, draft proofreading, and editing. RW contributed to the literature searches, ethics application, and data collection. All authors contributed to and have approved the final manuscript.

## Conflict of Interest Statement

The authors declare that the research was conducted in the absence of any commercial or financial relationships that could be construed as a potential conflict of interest.
